# Artificial light at night affects body mass but not oxidative status in free-living nestling songbirds: an experimental study

**DOI:** 10.1038/srep35626

**Published:** 2016-10-19

**Authors:** Thomas Raap, Giulia Casasole, David Costantini, Hamada AbdElgawad, Han Asard, Rianne Pinxten, Marcel Eens

**Affiliations:** 1 Department of Biology, Behavioural Ecology and Ecophysiology Group, University of Antwerp, Wilrijk, Belgium; 2 Department of Biology, Molecular Plant Physiology and Biotechnology Group, University of Antwerp, Antwerp, Belgium; 3 Department of Botany, Faculty of Science, University of Beni-Suef, Beni-Suef, Egypt; 4 Faculty of Social Sciences, Antwerp School of Education, University of Antwerp, Venusstraat 35, B-2000, Antwerp, Belgium

## Abstract

Artificial light at night (ALAN), termed light pollution, is an increasingly important anthropogenic environmental pressure on wildlife. Exposure to unnatural lighting environments may have profound effects on animal physiology, particularly during early life. Here, we experimentally investigated for the first time the impact of ALAN on body mass and oxidative status during development, using nestlings of a free-living songbird, the great tit (*Parus major*), an important model species. Body mass and blood oxidative status were determined at baseline (=13 days after hatching) and again after a two night exposure to ALAN. Because it is very difficult to generalise the oxidative status from one or two measures we relied on a multi-biomarker approach. We determined multiple metrics of both antioxidant defences and oxidative damage: molecular antioxidants GSH, GSSG; antioxidant enzymes GPX, SOD, CAT; total non-enzymatic antioxidant capacity and damage markers protein carbonyls and TBARS. Light exposed nestlings showed no increase in body mass, in contrast to unexposed individuals. None of the metrics of oxidative status were affected. Nonetheless, our study provides experimental field evidence that ALAN may negatively affect free-living nestlings’ development and hence may have adverse consequences lasting throughout adulthood.

The rapid increase of artificial light at night (ALAN), termed ‘light pollution’, is leading to a loss of darkness with largely unknown consequences for biodiversity, ecosystems and ecological and evolutionary processes^1^,^2^. This is likely to be problematic for many species as light has a strong biological relevance for the daily and annual rhythms of life^3^. Recently it is becoming clear that ALAN affects a wide variety of behavioural traits, such as reproduction, foraging, sleep and migration^4^, and has also physiological effects, including alterations in immune response, cortisol levels, testosterone levels and glucose metabolism^4^,^5^.

The impact of ALAN on individual status may be especially relevant when the organism is exposed in early life^6^, as the environment in which a young individual develops has profound, long-lasting and often irreversible consequences throughout the individual lifetime^7^,^8^,^9^,^10^. For example, in the wild, body mass of young birds at fledging is a good predictor of survival and recruitment because body energy reserves help individuals to cope with the adverse conditions of winter^11^,^12^,^13^,^14^. Given that ALAN can influence foraging behaviour of parents^15^ and sleep behaviour of nestlings^16^, it is plausible to expect an impact of ALAN on individual health and condition through its effects on body mass. However, it may impact individual status (other than body mass) also through other mechanisms, such as changes in oxidative status, especially since ALAN affects the immune response and cortisol levels^4^.

In recent years, ecologists have been studying antioxidants and oxidative damage in free-living organisms and have integrated principles of oxidative stress into several core evolutionary concepts, such as life-history trade-offs (e.g. survival versus reproduction), senescence and sexual selection^17^. Oxidative stress is a biochemical condition of the cell that occurs when there is an imbalance between pro-oxidants and antioxidants in favour of pro-oxidants leading to oxidative damage to biomolecules^18^,^19^. It is thought that oxidative stress is an important candidate mechanism underlying the effects of environmental changes on organism fitness because of its effects on growth^20^, fertility^21^, immune protection^22^ and cellular senescence^23^. It was shown in European shags (*Phalacrocorax aristotelis*) that fledglings with higher oxidative stress had a lower recruitment probability^24^ and in great tits (*Parus major*) that red blood cell resistance to oxidative stress predicted fledging success^25^. Laboratory work also showed that ALAN could influence the individual oxidative status^26^, as ALAN reduces melatonin^27^,^28^ which is an enhancer of antioxidant enzymes gene expression and known as a reactive oxygen species scavenger^29^,^30^,^31^,^32^. However, whether and how light pollution affects oxidative stress in the wild is still a major research gap^33^.

The variable nature of interactions among oxidative status biomarkers makes it very difficult to generalise the oxidative status from one or two measures^34^,^35^,^36^. The low correlations among biomarkers that are commonly found also imply that each biomarker reveals independent information on the oxidative status^34^,^35^,^36^. Moreover, one must take into consideration the fact that there is a vast array of antioxidant molecules that might respond to greater production of reactive species, as well as a large number of damage compounds can be produced^37^. In addition, either low or high antioxidant levels do not necessarily indicate whether damage is, or, is not occurring^19^, thus it is important to measure more than one type of marker of antioxidant protection along with markers of oxidative damage. To this end, in this study, we have relied on a multi-biomarker approach in order to obtain a better understanding of oxidative status.

In this study, we assessed for the first time the impact of disturbance induced by ALAN on body mass and multiple metrics of oxidative status (including antioxidant defences and oxidative damage) in nestlings of a free-living songbird, the great tit. The great tit is an important model species in evolutionary and environmental research. Although laboratory studies have often focused on one sex (e.g. ref. 38), we took into account that in birds, and especially in great tits, there may be sex-specific differences in oxidative status, growth rate^39^,^40^ and environmental sensitivity (reviewed in ref. 41), and we therefore used both male and female nestlings.

## Methods

### Study area and general procedures

Data were collected between 8 and 25 May 2015 in a resident suburban nest box population of great tits in the surroundings of Wilrijk, Belgium (51°9′44″N, 4°24′15″E). In 1997, nest boxes were installed and since then this free-living population has been continuously monitored^42^,^43^. Nest boxes made out of plywood with a metal ceiling were of standard size with outer dimensions of 120 × 155 × 250 mm (width × depth × height) and a nest box opening of 30 mm ø. During the breeding season, we checked nest boxes every other day, and every day when close to hatching, to determine laying date, clutch size, hatching day and fledging day. Nestlings were provided with a unique metal ring when they were between 11 and 13 days old (hatch day = day 1).

### Experimental procedure

While field studies on oxidative status (OS) often rely on single point measurements and experiments on free-living animals are often unfeasible^44^, we experimentally investigated effects of ALAN on OS using wild great tits and took repeated measurements as the latter is important for understanding physiological responses^44^ as well as to control for confounding variables (e.g. brood size) and variation generated by individuals. We randomly assigned 32 nests to one of the two treatment groups: a control (dark) and a light treated group. When nestlings were 13 days old, we collected a blood sample (≤150 μL) to determine their baseline levels of oxidative status and subsequently weighed them (0.1 g; digital balance; Kern TCB 200-1). We repeated this procedure after two nights when the nestlings were 15 days old, to assess changes in oxidative status and body mass. In the light group, nestlings were exposed to two consecutive nights of light (see *light treatment*), from day 13 to day 15.

Nests from the control and light group were always paired, primarily based on hatching date and similar brood size (7.0 ± 1.2 SD nestlings) and sampled on the same morning immediately after each other (between 8:00 and 12:00). The order of sampling the control and experimental nest(s) was kept the same within a pair but alternated between pairs so that there was no bias in the timing of sampling between the light and control group. Using a within individual and paired design is important as it eliminates many potential confounding variables^45^. In total, we obtained paired data on body mass and oxidative status from 16 nests in the control and 16 nests in the light group. From 115 nestlings in the control group and 109 in the light group we obtained body mass measurements. To get a representative sample on the oxidative status of each nest, we used blood samples of three nestlings for each nest, the heaviest, lightest and the median (*N* = 96), for further laboratory analyses. However, due to limitations in blood availability, sample size varies per oxidative status measurement (between 89–96; see Supplementary Table S1).

### Light treatment

In each nest box we placed a small LED light (15 mm × 5 mm, taken from a RANEX 6000.217 LED headlight, Gilze, Netherlands), which was standardized to produce 3 lux on the bottom of the nest box (ISO-Tech ILM 1335 light meter; Corby, UK), under the nest box roof lid. We have used this light system successfully in earlier studies on effects of ALAN on sleep behaviour^16^,^46^.

In the light-treated group, lights were turned on at 19:00 in the evening (about two hours before sunset) and turned off at 07:00 (about one hour after sunrise) the following morning. The control group had lights installed inside the nest box but these were always turned off, leaving these nests in a natural dark situation. Both groups were otherwise treated the same.

We based the length (two nights) and light intensity (3 lux) of our experimental treatment on previous laboratory studies because experiments as ours have not been done in the wild until now. Previous experimental studies on the physiological effect of ALAN^5^,^27^,^47^,^48^,^49^,^50^,^51^ used light intensities ranging from 0.05 to 5 lux and higher (100 lux; see for an overview^47^). In the laboratory, effects of light manipulations on melatonin levels were difficult to detect at lower light levels ≤0.5 lux and were more obvious using 1.5 and 5.0 lux in great tits^48^. We chose a light intensity of 3 lux with which we still expected to find differences in oxidative status but which was not too high so that parents would abandon their nests when the light was turned on^46^. Animals living in light polluted areas are exposed to similar and/or higher light intensities, especially outside nest boxes or cavities^46^,^49^,^50^. Because there is now a shift towards energy efficient broad spectrum light sources, such as LED for street lighting, we chose white LED light which has a broad spectrum^51^,^52^. Because of the energy efficiency of LED light there is no warming effect of the lights inside the nest boxes. Laboratory studies often use experimental periods of several weeks or months (e.g. refs 27 and 48), which is unfeasible with free-living developing nestlings. Short term light treatments (e.g. two nights of half an hour) have also been used in combination with high light intensity (450 lux)^38^. Two nights of ALAN may thus induce effects on oxidative status but limit the risk of any nest abandonment^46^.

### Laboratory analyses

With the use of PCR we determined the sex of nestlings^53^. We measured seven parameters of oxidative status^54^ in red blood cells and one in plasma. Using HPLC, we measured two molecular antioxidants in red blood cells: reduced glutathione (GSH) and oxidised glutathione (GSSG) after which we calculated the ratio GSH/GSSG, which is used as an index of redox state, with lower values indicating higher oxidative stress^55^. We also estimated the total non-enzymatic antioxidant capacity (TAC) and measured activity of three major antioxidant enzymes in red blood cells that differ in the way they protect cells against oxidative stress^54^,^56^: glutathione peroxidase (GPX), superoxide dismutase (SOD) and catalase (CAT). Finally, we measured protein carbonyls (marker of protein oxidation) in red blood cells, as well as thiobarbituric acid reactive substances (TBARS; marker of lipid peroxidation) in plasma, as markers of oxidative stress. Further details are given in the Supplementary material.

### Data analysis

For all statistical analyses we used R 3.1.2^57^. We performed a linear mixed effect analysis (LMM) on the effect of ALAN on nestling body mass (using the lme4 package^58^; see the Supplementary material about the justification of the use of LMM). As fixed effects, we entered “treatment” (control, light), “day” (13, 15), “sex”, “brood size” as well as the interaction between “treatment” and “day” and the three-way interaction “treatment”, “sex” and “day”. We used as random effect “bird identity” which was nested in “nest identity” which was nested in “pair” (bird identity:nest identity:pair) to control for the repeated measures and to take the experimental design into account (see experimental procedure).

We performed separate LMMs with the different metrics of oxidative status as dependent parameters. As fixed effects, we entered “treatment” (control, light), “day” (13, 15), “sex”, “brood size”, “body mass” and the three-way interaction “treatment”, “sex” and “day”. The same random structure was used as for the analysis on body mass (bird identity:nest identity:pair). In order to meet model assumptions the parameters GSH/GSSG, GSSG, TAC, GPX, CAT, SOD and protein carbonyls were square root transformed and TBARS was log transformed. P-values obtained by a stepwise backward regression are given in results (full model output is given in Supplementary Tables S2 and S3) and where applicable, Tukey HSD tests were used for post-hoc analyses (lmerTest^59^).

### Ethical statement

This study was approved by the ethical committee of the University of Antwerp (ID number 2014-45) and performed in accordance with Belgian and Flemish laws. The Belgian Royal Institute for Natural Sciences (Koninklijk Belgisch Instituut voor Natuurwetenschappen) provided ringing licences for authors and technical personnel.

## Results

**Figure 1:**
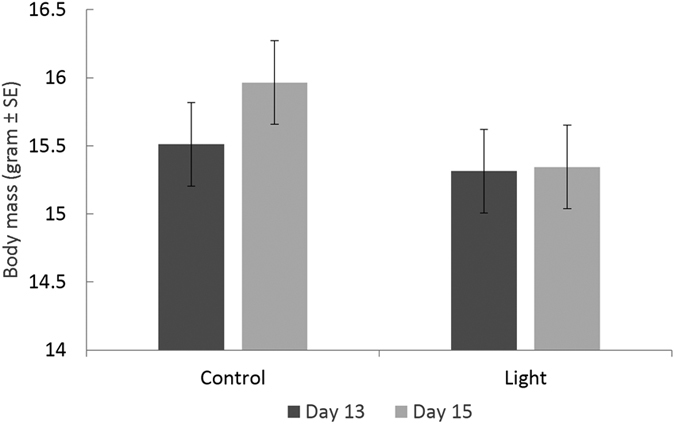
Effect of artificial light at night on nestling body mass. Estimates were obtained from linear mixed models with individual (*N* = 224) nested in nest (32) nested in pair as random factor (bird identity:nest identity:pair). Nestlings in the control group gained body mass between day 13 and day 15 (*t* = 7.41, *P* < 0.001) contrary to individuals in the light group whose body mass did not change (*t* = 0.047, *P* = 0.6).

Artificial light at night had a significant effect on nestling body mass (*F* = 7.209, *P* = 0.009, Fig. 1; full model output is given in Supplementary Table S2). Nestlings from the control group gained body mass between day 13 and day 15 (0.5 ± 0.06 gram, *t* = 7.14, *P* < 0.001), while body mass of individuals from the light group did not change (*t* = 0.47, *P* = 0.6). Males (*N* = 107) had on average larger body masses than females (*N* = 117; average of day 13–15 respectively 15.9 ± 0.230 and 15.2 ± 0.231 gram; *t* = 5.38, *P* < 0.001).

There was no sex dependent effect of artificial light on any of the oxidative status metrics and ALAN did not affect any of the oxidative status biomarkers measured (see Table 1 for full model results and Supplementary Table S3 for final models). Individuals in the control group had higher levels of GSH than those in the light group but because this was independent of time (no time:treatment interaction) it is not an effect of ALAN (0.6 ± 0.22 mmol/gram RBCs square root transformed; *t* = 2.54, *P* = 0.02). Males in the control group had lower levels of GSSG than those in the light group but this is not an effect of ALAN because it was independent of time (no time:treatment:sex interaction; see Supplementary Table S4 for estimates). There was no difference in GSSG between females in the control or light group (*t* = −1.01, *P* = 0.32). There was a small decrease in TAC, GPX and CAT over time (Supplementary Table S5). While there was no observable effect of ALAN on oxidative damage, there was a difference between sexes in TBARS (*F* = 7.914, *P* = 0.005; Supplementary Table S6) with an increase over time for males but not for females.

Four individuals did not fledge after the experiment, one individual from the control group and three from the light group. This difference was not significant (*X*^*2*^ = 1.131, *P* = 0.288).

## Discussion

Using a sophisticated experiment, in which the within-individual and paired design is likely to eliminate many confounding variables, we show that artificial light at night (ALAN) affects the development of free-living nestlings. ALAN had a significant negative effect on body mass gain of nestlings. Markers of oxidative status (OS) appeared to be unaffected by our short term light treatment.

We found that nestlings exposed to artificial light, contrary to those in the control group (who gained body mass in accordance with results from earlier studies, e.g. ref. 60), did not gain any body mass during a period of two days. An earlier study in our population showed that artificial light inside the nest box significantly increased nestlings’ activity as they started begging during the night while in the dark they hardly begged at all^16^. This implies that ALAN causes not only adults^16^,^46^ but also nestlings to be more awake and thus more active. There are two possible and not mutually exclusive explanations of how increased begging and or activity could lead to the observed lack of gain in body mass.

Firstly, this increased activity may lead to increased energy expenditure and a deterioration of body condition^61^,^62^,^63^. Rodriguez-Girones, *et al*.^64^ experimentally showed that increased begging (during the day) of ring dove (*Streptopelia risoria*) and magpie (*Pica pica*) nestlings can indeed lead to a decreased growth rate. If in our case the parents were unable to compensate for the increased energy expenditure, through an increased feeding rate or time the following day, it could explain why the chicks did not gain any body mass. It is not clear whether ALAN effectively enhances foraging and or food provisioning (see e.g. refs 15 and 65) and even if it does, it may not be sufficient to compensate energy loss of the nestlings. For example in adult blackbirds (*Turdus merula*) extension of foraging time did not affect body mass^66^. Moreover, in our study, LED lights were installed inside the nest box and this light does not create an environment outside the nest box with sufficient light to be used by the parents to extend their feeding time. It is therefore unlikely that our treatment could have extended feeding time of the parents. Using the same light treatment inside a nest box during a different experiment, we did not find any effect of ALAN on the time of entry of the female in the evening and although females did leave the nest box earlier in the morning^16^, it remains to be examined whether this time, when it is still dark outside, can effectively be used to feed nestlings.

Secondly, a long term experimental study on house sparrow (*Passer domesticus*) nestlings showed that costs of begging also include physiological costs besides affecting growth^62^. Therefore, an alternative explanation of how increased begging (and activity) could lead to reduced growth is provided by the energy allocation hypothesis. This hypothesis predicts that an increased maintenance cost reduces the proportion of energy spent on growth^67^,^68^, which was found to be true for nestlings of red-winged blackbirds (*Agelaius phoeniceus*)^69^. Increased begging (activity) could also increase metabolic demand thereby leading to oxidative stress^70^. In magpie nestlings, increased begging reduced growth but nestlings maintained their oxidative status^71^. Maintenance of oxidative status could therefore potentially reduce the proportion of energy spent on increases in body mass (see discussion on effects on OS below and Casagrande, *et al*.^72^).

Given that our short term light treatment already affected body mass gain in a period of two days, significant differences in body mass may possibly arise at fledging if nestlings are exposed during their entire development to ALAN. Although we did not find an effect on fledging with the current short light treatment, the effect of ALAN on early development of nestlings may not be limited to body mass, but could also affect metabolism, immune competence and sexual attractiveness in adulthood^7^. Moreover, body mass seems to be a good proxy for condition as heavier nestlings have higher nutritional reserves^73^ resulting in higher survivorship and recruiting probabilities^74^,^75^,^76^. Verhulst, *et al*.^77^ showed that body condition during early development (weight on day 16) correlates with the quality of the breeding habitat that the birds later occupy, which is another indication that a reduced body condition through artificial light could have effects into adulthood.

We did not find any effect of ALAN on oxidative status. There was no effect on molecular antioxidants or oxidative status as measured by the ratio between glutathione and reduced glutathione. Activity of antioxidant enzymes (GPX, CAT and SOD) were not affected nor was the total antioxidant capacity (TAC). Neither did we find any evidence that ALAN increased oxidative damage as measured by the amount of protein oxidation or lipid peroxidation.

Nonetheless, we could have expected our treatment to affect oxidative status. Firstly, our experimental treatment which consisted of two nights of light inside the nest box was sufficient to reduce body mass gain of nestlings. Secondly, our earlier studies showed that a single night and a lower light intensity (1.6 lux instead of 3 lux) had profound effects on sleep of adult great tits as well as on begging behaviour/sleep of nestlings^16^,^46^. ALAN therefore likely causes nestlings to be more active during the night and this may increase metabolic demand leading to oxidative stress^70^. Thirdly, the same treatment as we used here (3 lux during two nights) significantly increased haptoglobin while decreasing nitric oxide^47^, which are two important indicators of immunity, physiological condition and health state^78^,^79^.

There are several possible explanations as to why ALAN did not affect blood oxidative status in our experiment. (1) The great tits in our population are from a semi-urbanized area which could have already affected their oxidative status physiology (through adaptation to their environment), making them less susceptible to the stress induced by artificial light. (2) The length of our treatment might not be sufficient to affect blood oxidative status. (3) ALAN may have affected OS in other tissues than blood. (4) The increase in haptoglobin^47^ and reduced growth rate (lack of gain in body mass) may mask other effects of ALAN on OS.

Rural blackbirds have been shown to experience higher blood oxidative damage and higher baseline blood antioxidant defences compared to city blackbirds^80^. To show differences in OS between adult city and rural blackbirds, Costantini, *et al*.^80^ used, during an 11 month period, a repeated immune challenge and chronic disturbance which is a much longer and stronger disturbance than our two days of artificial light. It could be that a longer period of light exposure is necessary to induce oxidative stress (but see Methodology and below of why this is difficult to do with free-living nestlings). Differences between blood and liver oxidative stress measurements have been observed earlier in Brandt’s voles (*Lasiopodomys brandtii*) where experimental effects on SOD differed between serum and liver measurements^81^. A study using Mongolian gerbils (*Meriones unguiculatus*) also showed that measures of oxidative stress, antioxidant and damage are tissue dependent^82^. However, a recent study found that, except for oxidised glutathione (GSSG) and the ratio between GSH and GSSG, there was generally good qualitative and quantitative agreement between blood and tissue oxidative stress measurements (malondialdehyde, GSH, SOD, CAT, GPX, vitamin C and E)^83^. It is therefore unlikely that oxidative status in other tissues was affected by ALAN. In order to counteract increases in oxidative damage compounds, haptoglobin might have been elevated^84^. This might have eliminated the need to upregulate other antioxidants like GPX and CAT. Light exposed nestlings did not gain any body mass indicating a reduced growth rate, thus lowering metabolic activity, which might have masked to some extent any impact of ALAN on (some) metrics of oxidative status.

While we are the first to experimentally study the effect of ALAN on the oxidative status of free-living developing animals, our study has some limitations. Firstly, we used a cavity nesting bird as a model species because we can experimentally manipulate its light environment while the experimental manipulation of light conditions of open-nesting birds is much more difficult. Although the light intensity which we used (3.0 lux) may not always be experienced by nestlings of cavity nesting birds, behavioural changes have already been observed in adult male great tits using very low light intensities of 0.05 lux^48^. Future studies may build upon our results and examine effects on oxidative status and body mass using lower light intensities and longer experimental treatments. Nonetheless, we believe that our results offer insight in how ALAN affects free-living birds during development and that these results can also be relevant for other animals exposed to light pollution as they are exposed to similar and even higher light intensities^49^,^50^. Secondly, we used a short term light treatment as a long term treatment in a free-living population presents several practical and ethical issues. For example, while a short term light treatment of one night during winter may already deter adult birds from entering the nest box^46^, a long term artificial light treatment would then increase the risk of deterring adult birds which would have fatal consequences for the nestlings. Our design allowed us to sample nestlings twice. If we had taken samples from nestlings before the age of 13 days to obtain a longer treatment, the nestlings might have been too small to draw a sufficient amount of blood. Taking blood (and opening the nest box) from nestlings older than 15 days might induce early fledging in nestlings which would likely decrease their chances of survival. While we used a short term light treatment behavioural studies showed either no habituation of animals to ALAN or even larger effects as a consequence of long term exposure to light at night^48^,^85^. We showed that a short term light treatment affects nestlings’ body mass but subsequent studies may show if these effects are enhanced or ameliorated over longer treatments or if additional effects, also with regard to oxidative status, would appear.

## Additional Information

**How to cite this article**: Raap, T. *et al*. Artificial light at night affects body mass but not oxidative status in free-living nestling songbirds: an experimental study. *Sci. Rep.* **6**, 35626; doi: 10.1038/srep35626 (2016).

## Supplementary Material

Supplementary Information

## Figures and Tables

**Table 1:** Statistical output of the full mixed effect models, effect of artificial light on oxidative stress parameters.

		GSH	GSSG	GSH/GSSG	TAC	GPX	SOD	CAT	Protein carbonyls	TBARS
Sex: Treatment: Time	*F*	0.024	0.310	0.005	0.098	0.213	2.155	1.768	0.585	0.116
	*P*	0.877	0.579	0.946	0.754	0.645	0.144	0.185	0.445	0.733
Treatment: Sex	*F*	0.039	**6.400**	0.426	0.439	0.252	0.354	0.188	1.311	0.002
	*P*	0.843	**0.013**	0.516	0.508	0.616	0.553	0.665	0.254	0.961
Sex: Time	*F*	3.185	0.003	0.097	0.881	0.701	0.056	2.220	0.125	7.657
	*P*	0.078	0.958	0.756	0.349	0.404	0.814	0.138	0.724	0.006
Treatment: Time	*F*	1.479	0.376	1.735	2.811	0.237	0.288	1.973	0.189	0.761
	*P*	0.227	0.541	0.191	0.095	0.627	0.592	0.162	0.665	0.384
Treatment	*F*	5.444	0.885	0.000	1.785	0.535	0.337	0.378	0.018	0.754
	*P*	0.033	0.362	0.986	0.183	0.465	0.569	0.540	0.895	0.386
Sex	*F*	1.509	0.614	0.039	0.615	0.010	0.304	0.289	0.002	0.708
	*P*	0.223	0.436	0.843	0.434	0.922	0.582	0.591	0.964	0.401
Time	*F*	0.263	0.012	0.062	**9.564**	**11.416**	0.002	**22.191**	2.666	3.686
	*P*	0.609	0.915	0.803	**0.002**	**0.001**	0.964	**0.000**	0.104	0.056
Brood size	*F*	3.397	0.450	2.171	0.888	0.036	0.707	0.378	1.246	0.321
	*P*	0.076	0.508	0.147	0.351	0.851	0.407	0.541	0.266	0.571
Weight	*F*	1.920	3.430	0.038	0.007	0.346	3.542	0.234	0.143	0.074
	*P*	0.170	0.069	0.846	0.933	0.557	0.063	0.629	0.705	0.786

Linear mixed models with “bird identity” nested in “nest” nested in “pair” as random factor were used (bird identity:nest identity:pair). Significant values (*P* < 0.05) are depicted in bold, see Supplementary Table S1 for sample sizes per parameter (between 89–96 individuals). P-values obtained after a stepwise backward regression are mentioned in the main text (see also Supplementary Table S3).
