# Supplementary material for: Artificial light at night affects body mass but not oxidative status in free-living nestling songbirds: an experimental study
Source: Sci Rep. 2016 Oct 19;6:35626. doi: 10.1038/srep35626 (PMC5069498; doi:10.1038/srep35626)
Supplement: Supplementary Information [file srep35626-s1.doc]

**Supplementary Material**

**Artificial light at night affects body mass but not oxidative status in free-living nestling songbirds: an experimental study**

Thomas Raap ^1#*^

Giulia Casasole ^1#^

David Costantini ^1^

Hamada AbdElgawad ^2, 3^

Han Asard ^2^

Rianne Pinxten ^1, 4^

Marcel Eens ^1^

^1^Department of Biology, Behavioural Ecology and Ecophysiology Group, University of Antwerp, Wilrijk, Belgium

^2^Department of Biology, Molecular Plant Physiology and Biotechnology Group, University of Antwerp, Antwerp, Belgium

^3^Department of Botany, Faculty of Science, University of Beni-Suef, Beni-Suef, Egypt

^4^Faculty of Social Sciences, Antwerp School of Education, University of Antwerp, Venusstraat 35, B-2000, Antwerp, Belgium

^#^ Co-first author, these authors contributed equally to this work

* Corresponding author: [thomas.raap@uantwerpen.be](mailto:thomas.raap@uantwerpen.be)

**Laboratory analysis**

The choice of biomarkers was based on previous work on the effect of environmental light on the oxidative status [^1-4^](#_ENREF_1), as well as on literature showing the sensitivity of biomarkers to environmental stressors [^5^](#_ENREF_5). Markers of damage included protein carbonyls (well-established indicator of oxidative damage to proteins [^6^](#_ENREF_6)) and thiobarbituric acid reactive substances (TBARS, formed as a byproduct of lipid peroxidation [^7^](#_ENREF_7)). In terms of antioxidants, we measured the enzyme glutathione peroxidase (GPX) alongside the reduced (GSH) and oxidized (GSSG) forms of glutathione because GPX uses GSH to catalyse the breakdown of hydrogen peroxide and organic hydroperoxides into water and alcohol, respectively [^8^](#_ENREF_8). We have also measured the two antioxidant enzymes catalase (CAT) and superoxide dismutase (SOD), which detoxify cells from hydrogen peroxide (when it occurs at high concentrations) and superoxide anion, respectively [^7^](#_ENREF_7). Given that the antioxidant barriers also relies on non-enzymatic antioxidants, we have used the FRAP assay to estimate the non-enzymatic antioxidant [^7^](#_ENREF_7).

For detection of molecular antioxidants in red blood cells: reduced glutathione (GSH) and oxidised glutathione (GSSG), we used high-performance liquid chromatography with electro-chemical detection by a reversed-phase HPLC of Shimadzu (Shimadzu, ‘s Hertogenbosch, The Netherlands), following the protocol as described by [Sinha, et al^. 9^](#_ENREF_9). Concentrations of GSH and GSSG were expressed as micromole per gram fresh weight of red blood cells. The ratio between GSH/ GSSG was used as an index of redox state with lower values indicating higher oxidative stress [^10^](#_ENREF_10).

Activity of antioxidant enzymes, superoxide dismutase (SOD), catalase (CAT) and glutathione peroxidase (GPX) were determined from haemolysates of red blood cells. Red blood cells were homogenized by MagNALyser (Roche, Vilvoorde, Belgium) in 250 µl of extracting buffer (pH 7.4; 1.15% KCl and 0.02 M EDTA in 0.01 M PBS). All measurements were scaled down for semi-high throughput using a micro-plate reader (Multiskan RC plate reader type 351; Synergy Mx, Biotek Instruments Inc., Vermont, USA). SOD activity was determined by measuring the inhibition of nitroblue tetrazolium (NBT) reduction at 560 nm (ε_530_ = 12.8 mM^-1^ cm^-1^), following [Dhindsa, et al^. 11^](#_ENREF_11). CAT activity was measured following [Aeb^i 12^](#_ENREF_12), by monitoring the rate of decomposition of H_2_O_2_ (ε_240_ = 39.4 M^-1^cm^-1^). Activity of GPX was determined following [Drotar, et al^. 13^](#_ENREF_13) by measuring the decrease in NADPH absorbance measured at 340 nm and calculated from the 6.22 mM^−1^ cm^−1^ extinction coefficient. A modified ferric ion reducing antioxidant power (FRAP) assay was used to estimate the total antioxidant capacity (TAC) [^14^](#_ENREF_14). Homogenised red blood cells were mixed with the FRAP reagent, and the absorption was measured at 600 nm after 30 min. 6-Hydroxy-2,5,7,8-tetramethylchroman-2-carboxylic acid (Trolox) was used as the standard.

Finally, we measured protein carbonyls (marker of protein damage) in red blood cells, as well as thiobarbituric acid reactive substances (TBARS, marker of lipid peroxidation) in plasma, as oxidative stress markers. We followed the procedure explained in the “Protein Carbonyl Colorimetric Assay Kit” by Cayman Chemical's (Ann Arbor, MI, USA; see also [Levine, et al^. 15^](#_ENREF_15)) to measure protein carbonyl content after samples had been diluted with buffer extract to 2 mg protein ml−1. We estimated the concentration of TBARS as a measure of lipid peroxidation following [El-Shafey and AbdElgawa^d 16^](#_ENREF_16). Plasma was mixed with 0.5% (w/v) thiobarbituric acid (TBA) in 20% TCA. After incubation at 90°C for 45 minutes, samples were left to cool and then centrifuged at 10.000g. Absorbance was measured at 532, 600 and 450 nm and amount of MDA equivalents was calculated using the following formula: 6.45 × (A_532_ – A_600_) – 0.56 × A_450_ and expressed as nmol MDA equivalents g ^-1^ plasma.

**Data analysis**

Error terms are assumed independent in order to use the classic ANOVA and regression analysis. Since each bird/ nest was measured more than once we cannot assume independence between measurements. Mixed models were therefore used as they are a widely used technique to account for the non-independence between measurements in a dataset, by including random term effects into the regression equation. This ensures that significance of independent variables (fixed effects) is calculated while accounting for the non-independence of the measurements within the same individual/ nest [^17^](#_ENREF_17). Please see for averages of raw data Supplementary Table S7.

**Table S1** Number of individuals analysed for the different oxidative stress parameters. Individuals are from 16 nests in the control and 16 nests in the light group.

|  | **Control** | **Light** | **Total** |
| --- | --- | --- | --- |
| **GSH** | 48 | 48 | 96 |
| **GSSG** | 48 | 48 | 96 |
| **GSH/GSSG** | 48 | 48 | 96 |
|  |  |  |  |
| **GPX** | 45 | 44 | 89 |
| **CAT** | 46 | 43 | 89 |
| **SOD** | 47 | 45 | 92 |
| **TAC** | 47 | 45 | 92 |
|  |  |  |  |
| **Protein carbonyls** | 47 | 45 | 92 |
| **TBARS** | 48 | 48 | 96 |

**Table S2** Statistical output of the full mixed effect model, effect of artificial light at night on nestling weight. Linear mixed models with “bird identity” nested in “nest” nested in “pair” as random factor were used (bird identity:nest identity:pair). Significant values (*P* < 0.05) are depicted in bold, *N* = 224 individuals. P-values obtained after a stepwise backward regression are mentioned in the main text.

|  | *F* | *P* |
| --- | --- | --- |
| Treatment: Day: Sex | 0.177 | 0.675 |
| Treatment: Day | 21.472 | **<0.001** |
| Brood size | 2.085 | 0.160 |
| Sex | 35.193 | **<0.001** |
| Day | 27.853 | **<0.001** |
| Treatment | 1.131 | 0.675 |

**Table S3** Statistical output of the final mixed effect models, effect of artificial light on oxidative stress parameters. Linear mixed models with “nest” nested in “pair” as random factor were used (bird identity:nest identity:pair). See Supplementary Table S1 for sample sizes per parameter (between 89-96 individuals). P-values are obtained after a stepwise backward.

|  |  | **GSH** | **GSSG** | **GSH/GSSG** |  | **TAC** | **GPX** | **SOD** | **CAT** |  | **Protein carbonyls** | **TBARS** |
| --- | --- | --- | --- | --- | --- | --- | --- | --- | --- | --- | --- | --- |
| **Sex: Treatment: Time** | *F* | - | - | - |  | - | - | - | - |  | - | - |
|  | *P* | - | - | - |  | - | - | - | - |  | - | - |
|  |  |  |  |  |  |  |  |  |  |  |  |  |
| **Treatment: Sex** | *F* | - | 6.287 | - |  | - | - | - | - |  | - | - |
|  | *P* | - | 0.014 | - |  | - | - | - | - |  | - | - |
|  |  |  |  |  |  |  |  |  |  |  |  |  |
| **Sex: Time** | *F* | - | - | - |  | - | - | - | - |  | - | 7.914 |
|  | *P* | - | - | - |  | - | - | - | - |  | - | 0.005 |
|  |  |  |  |  |  |  |  |  |  |  |  |  |
| **Treatment: Time** | *F* | - | - | - |  | - | - | - | - |  | - | - |
|  | *P* | - | - | - |  | - | - | - | - |  | - | - |
|  |  |  |  |  |  |  |  |  |  |  |  |  |
| **Treatment** | *F* | 6.446 | 1.139 | - |  | - | - | - | - |  | - | - |
|  | *P* | 0.022 | 0.302 | - |  | - | - | - | - |  | - | - |
|  |  |  |  |  |  |  |  |  |  |  |  |  |
| **Sex** | *F* | - | 0.004 | - |  | - | - | - | - |  | - | 0.522 |
|  | *P* | - | 0.947 | - |  | - | - | - | - |  | - | 0.471 |
|  |  |  |  |  |  |  |  |  |  |  |  |  |
| **Time** | *F* | - | - | - |  | 10.598 | 11.042 | - | 22.726 |  | - | 3.772 |
|  | *P* | - | - | - |  | 0.001 | 0.001 | - | <0.0001 | | - | 0.053 |
|  |  |  |  |  |  |  |  |  |  |  |  |  |
| **Brood size** | *F* | - | - | - |  | - | - | - | - |  | - | - |
|  | *P* | - | - | - |  | - | - | - | - |  | - | - |
|  |  |  |  |  |  |  |  |  |  |  |  |  |
| **Weight** | *F* | - | - | - |  | - | - | 4.038 | - |  | - | - |
|  | *P* | - | - | - |  | - | - | 0.047 | - |  | - | - |

**Table S4** Results of post-hoc analyses for the interaction between treatment and sex for GSSG. Significant values (*P* < 0.05) are depicted in bold.

|  |  |  |  |  | estimate | se | *t* | *P* |
| --- | --- | --- | --- | --- | --- | --- | --- | --- |
| F | control | - | M | control | -0.2 | 0.098 | -1.83 | 0.07 |
| F | control | - | F | light | -0.1 | 0.096 | -1.01 | 0.32 |
| F | control | - | M | light | 0.1 | 0.100 | 0.73 | 0.47 |
| M | control | - | F | light | 0.1 | 0.100 | 0.82 | 0.42 |
| M | control | - | M | light | 0.3 | 0.104 | 2.41 | **0.02** |
| F | light | - | M | light | 0.2 | 0.098 | 1.74 | 0.09 |

**Table S5** Results of post-hoc analyses for the difference in TAC, GPX and CAT between day 13 and 15 independent of light treatment. Estimates are given and *t* and *P* values for the difference between days.

| Parameter | Day | estimate | se | *t* | *P* |
| --- | --- | --- | --- | --- | --- |
| TAC | 13 | 0.31 | 0.01 |  |  |
|  | 15 | 0.29 | 0.01 | 3.26 | 0.001 |
| GPX | 13 | 0.07 | 0.00 |  |  |
|  | 15 | 0.06 | 0.00 | 3.32 | 0.001 |
| CAT | 13 | 0.61 | 0.02 |  |  |
|  | 15 | 0.53 | 0.02 | 4.77 | <0.0001 |

**Table S6** Results of post-hoc analyses for the interaction between sex and treatment for TBARS. Significant values (*P* < 0.05) are depicted in bold.

|  |  |  |  |  | estimate | se | *t* | *P* |
| --- | --- | --- | --- | --- | --- | --- | --- | --- |
| F | 13 | - | M | 13 | 0.00 | 0.03 | 1.40 | 0.163 |
| F | 13 | - | F | 15 | 0.00 | 0.03 | 0.63 | 0.527 |
| F | 13 | - | M | 15 | -0.10 | 0.03 | -1.86 | 0.064 |
| M | 13 | - | F | 15 | 0.00 | 0.03 | -0.81 | 0.418 |
| M | 13 | - | M | 15 | -0.10 | 0.03 | -3.28 | **0.001** |
| F | 15 | - | M | 15 | -0.10 | 0.03 | -2.48 | **0.014** |

**Table S7** Raw average for metrics of oxidative status. Average obtained from raw data are given with their standard deviation per treatment group, control or light exposed nestlings.

|  | **Control** |  | **Light** |  |
| --- | --- | --- | --- | --- |
|  | **Day 13** | **Day 15** | **Day 13** | **Day 15** |
| **GSH** μmol/g fresh weight | 2.808 ± 1.388 | 3.033 ± 1.297 | 2.398 ± 1.042 | 2.314 ± 1.263 |
| **GSSG** μmol/g fresh weight | 0.884 ± 1.272 | 0.912 ± 1.274 | 0.653 ± 0.731 | 0.708 ± 0.707 |
| **GSH/ GSSH** | 16.877 ± 44.972 | 30.304 ± 107.009 | 11.276 ± 13.574 | 10.078 ± 13.134 |
| **TAC** µmol trolox/g fresh weight | 0.126 ± 0.106 | 0.094 ± 0.065 | 0.103 ± 0.087 | 0.088 ± 0.061 |
| **GPX** μmol NADPH/mg prot/min | 0.005 ± 0.005 | 0.004 ± 0.004 | 0.005 ± 0.005 | 0.004 ± 0.004 |
| **CAT** μmol H2O2/mg prot/min | 0.462 ± 0.371 | 0.335 ± 0.252 | 0.42 ± 0.363 | 0.32 ± 0.217 |
| **SOD** U/mg prot/min | 0.414 ± 0.368 | 0.396 ± 0.152 | 0.43 ± 0.224 | 0.409 ± 0.15 |
| **Protein carbonyls** nmol/mg prot | 1.663 ± 1.993 | 1.856 ± 2.04 | 1.571 ± 1.765 | 1.832 ± 1.716 |
| **TBARS** nmol MDA/ g plasma | 66.398 ± 15.575 | 68.025 ± 19.445 | 85.37 ± 159.466 | 69.32 ± 21.785 |

References

1 Baydas, G., Ercel, E., Canatan, H., Donder, E. & Akyol, A. Effect of melatonin on oxidative status of rat brain, liver and kidney tissues under constant light exposure. *Cell Biochem. Funct.* **19**, 37-41, doi:10.1002/cbf.897 (2001).

2 Cruz, A. *et al.* Effect of melatonin on cholestatic oxidative stress under constant light exposure. *Cell Biochem. Funct.* **21**, 377-380, doi:10.1002/cbf.1046 (2003).

3 Hardeland, R., Coto-Montes, A. & Poeggeler, B. Circadian rhythms, oxidative stress, and antioxidative defense mechanisms. *Chronobiol. Int.* **20**, 921-962, doi:10.1081/Cbi-120025245 (2003).

4 Ashkenazi, L. & Haim, A. Effect of light at night on oxidative stress markers in Golden spiny mice (*Acomys russatus*) liver. *Comp. Biochem. Physiol. A Mol. Integr. Physiol.* **165**, 353-357, doi:10.1016/j.cbpa.2013.04.013 (2013).

5 Costantini, D. *Oxidative stress and hormesis in evolutionary ecology and physiology*. (Springer, 2014).

6 Dalle-Donne, I., Rossi, R., Giustarini, D., Milzani, A. & Colombo, R. Protein carbonyl groups as biomarkers of oxidative stress. *Clin. Chim. Acta* **329**, 23-38, doi:10.1016/S0009-8981(03)00003-2 (2003).

7 Halliwell, B. & Gutteridge, J. M. *Free radicals in biology and medicine*. 4th edn, (Oxford University Press, USA, 2007).

8 Yu, B. P. Cellular defenses against damage from reactive oxygen species. *Physiol. Rev.* **74**, 139-162 (1994).

9 Sinha, A. K. *et al.* Anti-oxidative defences are modulated differentially in three freshwater teleosts in response to ammonia-induced oxidative stress. *PLoS One* **9**, e95319, doi:10.1371/journal.pone.0095319 (2014).

10 Jones, D. P. Redefining oxidative stress. *Antioxid. Redox Signal.* **8**, 1865-1879, doi:10.1089/ars.2006.8.1865 (2006).

11 Dhindsa, R. S., Plumbdhindsa, P. & Thorpe, T. A. Leaf Senescence - Correlated with Increased Levels of Membrane-Permeability and Lipid-Peroxidation, and Decreased Levels of Superoxide-Dismutase and Catalase. *J. Exp. Bot.* **32**, 93-101, doi:DOI 10.1093/jxb/32.1.93 (1981).

12 Aebi, H. in *Methods Enzymol.* Vol. Volume 105 (ed Packer Lester) 121-126 (Academic Press, 1984).

13 Drotar, A., Phelps, P. & Fall, R. Evidence for Glutathione-Peroxidase Activities in Cultured Plant-Cells. *Plant Sci.* **42**, 35-40, doi:Doi 10.1016/0168-9452(85)90025-1 (1985).

14 Benzie, I. F. & Strain, J. J. The ferric reducing ability of plasma (FRAP) as a measure of "antioxidant power": the FRAP assay. *Anal. Biochem.* **239**, 70-76, doi:10.1006/abio.1996.0292 (1996).

15 Levine, R. L. *et al.* in *Methods Enzymol.* Vol. Volume 186 464-478 (Academic Press, 1990).

16 El-Shafey, N. M. & AbdElgawad, H. Luteolin, a bioactive flavone compound extracted from Cichorium endivia L. subsp divaricatum alleviates the harmful effect of salinity on maize. *Acta Physiol. Plant.* **34**, 2165-2177, doi:10.1007/s11738-012-1017-8 (2012).

17 Fitzmaurice, G., Laird, N. & Ware, J. *Applied longitudinal analysis*. (Wiley, 2004).
